# Fail to Prepare and you Prepare to Fail: the Human Rights Consequences of the UK Government’s Inaction during the COVID-19 Pandemic

**DOI:** 10.1007/s41649-020-00151-1

**Published:** 2020-11-02

**Authors:** Rhiannon Frowde, Edward S. Dove, Graeme T. Laurie

**Affiliations:** School of Law, University of Edinburgh, Edinburgh, UK

**Keywords:** COVID-19, United Kingdom, Human rights, Law, Preparedness

## Abstract

As the sustained and devastating extent of the coronavirus disease 2019 (COVID-19) pandemic becomes apparent, a key focus of public scrutiny in the UK has centred on the novel legal and regulatory measures introduced in response to the virus. When those measures were first implemented in March 2020 by the UK Government, it was thought that human rights obligations would limit excesses of governmental action and that the public had more to fear from unwarranted intrusion into civil liberties. However, within the first year of the pandemic’s devastation in the UK, a different picture has emerged: rather than through action, it is governmental *inaction* that has given rise to greater human rights concerns. The UK Government has been roundly criticized for its inadequate response, including missteps in decision-making, delayed implementation and poor enforcement of lockdown measures, abandonment of testing, shortages of critical resources and inadequate test and trace methods. In this article, we analyse the UK Government’s missteps and compare them with published international guidance; we also contrast the UK’s decisions with those taken by several other countries (including the devolved administrations within the UK) to understand how its actions and inactions have contributed to unfavourable outcomes. Using an analytical perspective that demonstrates how human rights are both a protection from the power of the state and a requirement that governmental powers are used to protect the lives, health and wellbeing of citizens, we argue that the UK Government’s failure to exercise their powers competently allowed the virus to spread without ensuring the country had the means to manage a high case load. This abject failure has led to one of the highest rates of deaths per capita worldwide. We offer several lessons that can be learnt from this unfortunate, but preventable, situation.

## Introduction

The coronavirus disease 2019 (COVID-19) pandemic is an ongoing global crisis caused by the SARS-CoV-2 virus (severe acute respiratory syndrome coronavirus 2). First formally identified in December 2019 in Wuhan, China, the virus was declared a public health emergency of international concern (PHEIC) by the World Health Organization (WHO) on 30 January 2020, and then declared a pandemic on 11 March 2020 (World Health Organization 2020d). The virus is highly contagious, with potential to cause severe illness or death; indeed, as of the time of writing, COVID-19 has infected tens of millions of people worldwide, and, factoring in the suspected large-scale undercount across the globe, the death toll has exceeded one million (and by some estimates, is closer to two million) (The Economist 2020b). Given the high risk to life, governments around the world have enacted a raft of measures to try to stop its spread. This has significant implications for human rights, including the rights to life, liberty and security of a person (World Health Organization 2020b).

COVID-19 hit the United Kingdom (UK) with force beginning in March 2020, though the virus was undoubtedly circulating across the country in the months prior. On 11 March, the day the WHO declared COVID-19 a pandemic, there were 70 reported cases in the UK in the preceding 24 hours; by 31 March, there were 2726 cases in the preceding 24 hours (Public Health England 2020b). In the early stages of the pandemic, the focus of much public scrutiny was on the human rights implications (Laurie 2020), particularly of the newly introduced legislative and regulatory measures, such as the Coronavirus Act 2020, and other subsequent legislation (Hosali 2020; Parpworth 2020). Concern was expressed about the potential inference with freedoms and civil liberties, particularly freedom of movement. However, what has transpired in the subsequent months is not so much a picture of interference with human rights through governmental action, but rather a scene of extensive interference through governmental *inaction*. This is most clear when the UK’s response is compared to that of other countries and the results they have achieved.

It is now evident that some countries are managing the ongoing crisis relatively well, with notably fewer cases and deaths. The UK is not among these. It has experienced one of the worst-case outbreaks in the first wave of infection, making it one of the worst affected in the world at the time of writing this article^1^ (Our World in Data 2020d). In July 2020, it was reported that England had the highest excess death rate in Europe over first half of the year (Office for National Statistics 2020b). Moreover, these figures are considered to be an underestimate due to severely limited testing capacity at the start of the pandemic in March and April (Perrigo 2020). The UK has one of the highest rate of deaths per capita of any large country (Our World in Data 2020b)—currently 62 confirmed deaths per 100,000 people. Public opinion and indeed consensus have been reached already, across the political divide, that something has gone terribly wrong (Devlin and Connaughton 2020). In this article, we argue that that something, and that wrong, have been the inaction and incompetence of the UK Government, which in consequence has likely breached the human rights of its citizens.

In what follows, we first explore how the UK Government’s approach in the early stages of the pandemic differed from international guidance and actions taken by other countries, as well as some of the devolved administrations within the UK (here we focus on the actions taken by the Scottish Government). As countries were shutting down their borders and issuing stay-at-home orders as soon as the severity of the virus was known, the UK withheld implementing a lockdown until some weeks later; this time lag likely led to thousands of deaths that could have been prevented. When countries were entering the global race to secure critical resources, the UK did not follow suit, leading to shortages of personal protective equipment (PPE), testing kits and ventilators, for both frontline healthcare workers but also social care providers for the elderly (BBC 2020f). Moreover, the UK did not screen international arrivals at ports of entry (having only enacted a 14-day quarantine rule for international arrivals in June 2020), including at London-Heathrow—one of the world’s busiest airports—and it abandoned mass testing and tracing measures, contrary to international guidance. To date, the UK continues to struggle with implementing a robust test and trace system, including through both testing centres and its COVID-19 smartphone app that runs in England and Wales (Wise 2020; Griffin 2020).

Following this account, we examine the consequences of the UK Government’s inaction in terms of its negative impact on the citizenry. Delayed implementation of physical distancing measures and abandonment of community-wide testing allowed uncontrolled spread of the virus, sharply increasing the number of infections. Without the capacity to manage this surge, the healthcare system was nearly overwhelmed in April, and measures to lessen the burden on the NHS led to greater harms. Among the worst affected in the population have been health and social care workers and the elderly, and there has been a stark disproportionate impact on Black, Asian and minority ethnic (BAME) populations, not to mention other groups who are vulnerable because of wider structural injustices.

Human rights require governments to protect their citizens’ health and wellbeing, and thus in the final main section, the consequences of all of this inaction are explored in light of these duties. While more stringent measures, as implemented in other countries, may cause greater limitations of individual rights, such as liberty and privacy, we argue herein that inaction may equally be found to have caused human rights infringements through, for example, greater loss of life and insecurity of the person.

Contrary to the messages of success promoted by the UK Government, there is evidence that delays, errors and general incompetence have contributed to a substantial humanitarian and human rights disaster.

## In What Ways did the UK Fail to Respond Adequately to the Advent of COVID-19 relative to International Guidance and Action in other Countries?

When the first COVID-19 cases emerged in East Asia in January 2020, the UK Government stated that the country was ‘well prepared’ (BBC 2020a). As the virus spread to Iran and Italy in the subsequent weeks, this message was reiterated. And, as the virus continued to then spread rapidly and devastatingly across Europe in early March, the plan to ‘contain, delay, research and mitigate’ was supported by all of the UK nations, being England, Wales, Scotland and Northern Ireland. However, within weeks, it became necessary to abandon this plan as cases surged and the UK found itself overwhelmed and underprepared (Scally et al. 2020). This led to the nations within the UK taking divergent approaches to tackle the pandemic. We unpack the UK-wide failures in turn but first briefly describe the system of devolved powers in the four nations’ kingdom.

Responsibility for legal and practical responses to infectious diseases is devolved from the central government to the principal four UK jurisdictions, being England, Wales, Scotland and Northern Ireland. In accordance with this, the Coronavirus Act 2020 conferred powers to take emergency action in response to the pandemic to devolved ministers. While the responses were largely coordinated at the initial stages, as the UK as a whole went into lockdown, the policies of each of the devolved nations diverged as the situation developed. Statistical data on the impact of the virus suggest that England has experienced an unusually high mortality and infection rate in comparison to other parts of the UK (Dickie and Burn-Murdoch 2020). Not all matters are devolved, however, as the four nations do not operate within silos, and a necessary reliance on Westminster remains where decisions must be taken at a UK national level. For example, some key policies that undoubtedly significantly influence infection rates and distribution, such as border and immigration control, remain reserved matters decided by Westminster alone. The track and trace system, highlighted by all governments as being key to limiting infections and preventing a second wave, is most effective when interoperable between all four nations; however, the politics of devolution challenge this possibility (Walker 2020; Parker 2020). Furthermore, the lack of information from Westminster on the effectiveness of the so-called NHS track and trace system has drawn the criticism that it has obstructed the individual UK nations from meeting their own public health objectives. Therefore, the UK’s failures may be observed as a whole, despite some degree of variation between the nations.

### Early Failures—Lack of Preparedness

When considering the extent of the UK Government’s inaction, we should start by looking at action plans prior to the current pandemic. Most countries have pandemic response plans, as they have duties to protect their populations and pandemics can have devastating consequences for human life and national economies. When effective, these plans are continuously reviewed and updated based on emerging evidence and international guidance. The UK had a pandemic response plan in place—but this was much more evident on paper than in reality. The limitations of the UK’s preparedness were known for years prior to COVID-19. For example, significant weaknesses in the UK’s emergency preparedness, resilience and response (EPRR) plan were highlighted in 2016, yet the necessary remedial steps do not appear to have been taken before the advent of COVID-19 (Nuki and Gardner 2020). The simulation exercise carried out by NHS England in October 2016, ‘Exercise Cygnus’, was conducted under the auspices of Public Health England and modelled an influenza pandemic; the leaked report showed that the exercise found that the NHS in England would collapse from a lack of resources and that a shortage of ventilators and capacity for disposal of the deceased would present serious challenges. The NHS’s surge capacity was shown to be of serious concern, with a shortage of PPE and ICU beds (Lambert 2020; Smyth 2016).

The same team that ran Exercise Cygnus is currently tracking and responding to the COVID-19 pandemic. Despite acute awareness of these weaknesses, it was precisely this lack of surge capacity that required measures to ease the burden on the UK’s health services, rather than focusing primarily upon supporting the health and wellbeing of the UK population (Nuki and Gardner 2020). Poor pandemic planning also led to delays in purchasing essential equipment and tests and the issuance of mixed messages about public health practices. Furthermore, the UK Government has been accused of not having the capacity to deal with a pandemic of this scale and severity as a result of spending cuts on healthcare over the past years in the name of austerity following the global financial crisis of 2007–2008 (Woodcock 2020b).

### A Comparative Approach to Pandemic Responses^2^

As mentioned above, the WHO declared the virus a PHEIC on 30 January 2020 (World Health Organization 2020d), and in early March 2020, NHS England and NHS Improvement declared its first ever level 4 critical incident (Discombe 2020). This meant that the response to the virus would be coordinated at a national level. Despite these early warnings, the UK declined to join a European scheme to source personal protective equipment (PPE). The UK’s focus on leaving the European Union led it to miss eight meetings about the virus, between 13 February and 30 March, with EU heads of state and health ministers (Tolhurst 2020). This, in turn, led to a missed deadline to participate in a common purchase scheme for critical health supplies (Boffey 2020; BBC 2020e). This would prove to be a serious error, creating shortages of critical resources for frontline health workers. All too soon, NHS bosses were warning of PPE shortages and a lack of surge capacity (Calvert et al. 2020; Foster and Neville 2020). In late February 2020, a worst-case scenario report from the government was leaked, suggesting that 500,000 Brits could die if the UK followed its prevailing course of action—this was to avoid lockdown, resist scaling up of testing and tracing regimes and not to implement any screening of international arrivals at ports of entry. The UK Government did not act on these concerns (Lintern 2020) until several weeks later.

Throughout March 2020, lives were continuing as normal in the UK, while all around, its neighbours were shutting down due to a growing awareness that the virus was airborne and spread through contact and physical proximity. In early-to-mid March, for example, Italy, France and Spain went into lockdown. At this stage, the UK was still managing some form of contact tracing, but continuance of mass public gatherings allowed the virus to continue to spread (Perrigo 2020)—indeed, large concerts and sporting activities went ahead well into March. While on 5 March, Greece was closing its schools, following the path of Iran and Italy (Jones et al. 2020), Boris Johnson, the UK Prime Minister, attended a Six Nations Rugby match only 2 days later with 82,000 closely packed spectators (Archer 2020). When France banned large events and began to introduce stricter social distancing measures (Landler and Castle 2020), and Ireland cancelled its St. Patrick’s Day parade on 9 March (BBC 2020b), the UK Government did not find the need for a nationwide lockdown. Instead, the Cheltenham Festival went ahead, attracting crowds of over 250,000 people over the course of 4 days (Morris 2020). When the WHO declared a pandemic on 11 March (BBC 2020c), and Madrid closed its schools as it became the epicentre of Spain’s coronavirus crisis (Jones 2020b), the UK welcomed 3000 Atletico Madrid fans flying in from Spain to Liverpool (Atkinson 2020).

Despite international advice to prioritize testing, the UK abandoned its contact tracing strategy on 12 March, on the basis that the UK did not have the capacity to maintain the mass testing strategies that were being implemented in other countries. The strategy seemed to be allowing the virus to spread through the population, so that a form of ‘herd immunity’ would be developed (Shields 2020; Scally et al. 2020). Following this, the WHO stated that ‘tracing every contact must be the backbone of the response in every country’ (World Health Organisation 2020a) yet mass testing would not resume in the UK until many months after. Despite the UK having shifted to the ‘delay’ phase, the order to physically distance did not follow for another 4 days. Even so, these measures were not legally binding, nor were businesses required to close. Moreover, despite Europe being declared by the WHO as the epicentre of the pandemic in March (World Health Organization 2020c), the UK continued to freely allow arrivals into the country from known hotspots, including Wuhan, northern Italy and Iran (Woodcock 2020a).

The lack of enforced physical distancing measures and continuance of mass public gatherings led directly to cases rising exponentially in the UK. It eventually became obvious that indoor spaces, such as offices, schools, restaurants and pubs, would have to close. Yet, the UK Government representatives continued to claim that the country was managing well, suggesting that PPE and capacity shortages had been resolved (Horton 2020). Behind these claims, however, the UK had downgraded its guidance on PPE, from level 4 to level 3 (Scally et al. 2020). It was discharging elderly patients from hospitals into care homes without testing or quarantining in order to increase the number of available hospital beds for the coming waves of infected patients (Rushton and Barnes 2020). This proved to be catastrophic, as infected elderly patients further spread the virus into care homes, leading to mass casualties across the country.

The UK only entered lockdown on 23 March (Brown 2020), nearly 2 weeks after most of Europe had done so. By this point, there were 967 recorded cases, but due to the lack of testing, it is likely this in fact was much higher. At that stage, the UK had yet to reach its own target of 25,000 tests per today (Panjwani 2020). By 7 April, the UK had conducted only a fifth of the number of tests as Germany and the death toll was over 10,000 (Hall and Buck 2020). By 18 April, it was recognized that PPE was running out (Milligan 2020; Siddique 2020; Blackall 2020). The death toll continued to rise exponentially, comprised in large part of deaths in care homes (O'Dowd 2020). Despite this, the government rejected a plan to lock down care homes (Booth 2020); meanwhile, 10% of these deaths were among frontline health workers, many of whom had repeatedly voiced concerns about a lack of PPE (Torjensen 2020).

The UK Westminster Government began to exit its lockdown on 11 May, against the WHO advice that it should not do so without a fully functioning test and trace system (Wood 2020); arguably, the consequences of this have been evidenced with the second wave that hit the UK in September. Moreover, this exit from lockdown differed from approaches taken in the governments of the nations across the UK. For example, the Scottish Government has been somewhat more cautious about emerging from lockdown, implementing a four-phase approach that has sought to avoid reopening establishments and social gatherings at quick pace (Deputy First Minister 2020). Whereas, to take one example, pubs and restaurants could reopen in England on 4 July with physical distancing measures in place (in principle, though whether this was enforced is open to question), this did not happen in Scotland until 15 July; whereas gyms reopened in England on 25 July, they did not in Wales until 10 August and in Scotland until 31 August. Whereas wearing a face covering became mandatory in shops and supermarkets in England only from 24 July, they were mandatory in Scotland since 10 July (in all instances subject to relatively loose ‘legitimate exemption’ criteria). These different approaches, not surprisingly, have led to political tensions, with some commentators calling for a more unified approach across the country and with others warning that devolved administrations (which have responsibility over heath and public health) following the UK Government’s approach—which applies in respects to England only, with the notable exception of border control—would exacerbate rising case levels and deaths (Marshall 2020). As noted above, as of the time of writing, the UK is, as is much of Europe, experiencing a second wave of the virus and consequently, we again are seeing the four nations taking different approaches to limit the rise in cases and fatalities.

It was not just the devolved administrations that decided to take a more cautious approach to emerging from lockdown; various leading medical bodies expressed concern over the risks of easing the lockdown too soon, and the number of cases and deaths were still too high to allow for effective testing and tracing (English and Rae 2020). The track and trace smartphone app developed by NHSX in England faced a significant number of teething problems and was only rolled out in late September (and continues to face problems) (Bosley 2020; Wise 2020; Griffin 2020; Wright and Bodkin 2020); and the so-called NHS Test and Trace programme in England has repeatedly been unable to reach up to a third of persons (Warrell and Hughes 2020; Marsh 2020). Indeed, *The Guardian* newspaper reported in July 2020 that in areas with the highest infection rates in England, the proportion of close contacts of infected people being reached was far below 80%, the level the government’s scientific advisers say is required for test and trace to be effective (Halliday 2020); in Luton, only 47% of at-risk people were contacted by test and trace. Worse, the UK Government itself admitted the so-called NHS Test and Trace programme it launched for England on 28 May was unlawful, as it violated the Data Protection Act 2018 in failing to conduct a data privacy impact assessment (DPIA) (Manthorpe 2020). Other routes for dealing with the pandemic, such as vaccines or effective medication, are unavailable or still in trial phases (Gartner et al. 2020); there is no guarantee either an effective (or widely available) vaccine or treatment will be developed, much less in the near future. Already, following England’s exit from the 23 March lockdown, there have been surges across all areas of the country, requiring local lockdowns, such as in the city of Leicester in late June (BBC 2020g) and more recently in Cardiff and Swansea, and intermediate lockdowns, such as requiring university students in Manchester to self-isolate ‘with immediate effect’ (BBC 2020h). This is in contrast to other countries that are reporting declines in numbers of new cases, despite having implemented their lockdown exit plans and beginning to emerge from them.

As this account outlines, the UK Government often departed from and fell behind international guidance and action, influencing not only the number of confirmed cases in the country but also the country’s ability to manage these, leading to a staggeringly high case fatality rate (CFR) in comparison with the global average (Our World in Data 2020a). As we will see, the UK Government’s response all stands apart from those taken by other countries, including those who might otherwise be seen as lacking the resources and robust public health infrastructure to tackle a pandemic.

### Global Responses to COVID-19: Where and How did they Differ?

Among those large countries with the lowest number of cases and deaths to date are Vietnam, Thailand, Laos, Cambodia, Taiwan, Singapore, Sri Lanka, South Korea and New Zealand (Our World in Data 2020a; Our World in Data 2020c; Bremmer 2020). As we can see, success is not dictated necessarily by GDP per capita; rather, it has been more a result of robust planning and preparedness (including test and trace programmes), government coordination across vertical and horizontal levels (i.e. between central and local governments and across ministries), strict border controls, political commitment and close involvement with competent public health experts.

Vietnam, recognizing early on its medical system could be overwhelmed, opted for strong prevention early on as soon as reports emerged from China, leading it to have reported around 1100 cases (averaging 11 cases per million people) and only 35 confirmed deaths as of the time of writing (Vietnam Ministry of Health 2020). Although it may be that these low numbers are linked to limited testing and challenges in the attribution of cause of death, it is safe to assume the numbers are significantly low given that there is no reporting of health services being overstretched, and a similar picture seems to have endured across much of Southeast Asia. Sri Lanka, to take another example, went into swift and strict lockdown, shutting down all ports of entry to international arrivals and mandating face mask wearing in public spaces, and, to date, has reported only 13 deaths (Epidemiology Unit 2020).

Most remarkably perhaps, Taiwan, despite not shutting down the country and its economy, and by implementing comprehensive contact tracing through SIM cards to ensure compliance with quarantine requirements, has only reported just over 500 cases and 7 confirmed deaths. Additionally, the government’s response, setting strict precautionary measures such as taking temperatures and providing sanitiser, has been largely regarded as credible (Hancocks 2020).

South Korea reacted rapidly and early on, scaling up testing even before its number of confirmed cases exceeded a hundred incidences, to a point where they even had sufficient resources to export testing kits (Ferrier 2020). Again, even without a lockdown, the cases are relatively low at 462 per million (Taiwan Centers for Disease Control 2020).

In common with these countries were populations who were willing to make trade-offs of commerce and movement, and, in some cases, privacy, for their safety. Outside South and East Asia, despite a very different political system, New Zealand performed well. New Zealand shut its borders less than 3 weeks after their first case, and a week later implemented a strict lockdown in which individuals could only socialize within their household. At the time of writing, it has reported only 367 cases per million and a CFR of around 1.7%.

## What are the Main Consequences of this in Terms of Negative Impact on the Citizenry?

A common characteristic in all of the countries with low case levels and death tolls is that they all reacted quickly, decisively and robustly to the pandemic. Important measures included thorough pandemic planning (as well as implementation of recommendations from reports), adequate surge capacity and resources, rapid and well-communicated responses such as implementation of stay home orders and comprehensive testing and tracing systems. In contrast, the UK Government’s decision not to take decisive action until late March 2020 allowed shortages of vital health equipment, including tests, to become a critical issue.

In short, the UK Government downplayed the severity of the pandemic until it was too late. Even when modelling suggested there could be 500,000 deaths, the Government maintained they were ‘well prepared’. In fact, the UK was unprepared for a pandemic of this severity, fighting a high-risk virus with a plan best suited for a low-risk flu. By allowing the virus to spread through the country in their ‘delay’ phase, the UK placed the lives of its citizens directly at risk. Countries which were willing to react quickly and implement admittedly stringent measures kept many of their citizens from contracting the virus. Indeed, in some countries, such as South Korea, a lockdown was not required because of good planning, clear public communication and exceptionally thorough tracking and tracing. For all countries that can be seen to have managed the initial wave of COVID-19 well, their actions have all relied on effective contact tracing, with the result that they continue to be capable of ensuring outbreaks remain localized (Sridhar 2020).

### Confirmed Cases and Deaths

The UK’s infection cases per million is high, but more concerning is its CFR, which is among the worst in the world. By shifting from the ‘contain’ to the ‘delay’ phase and abandoning testing in the early stages, the continuance of public gatherings in the UK allowed the virus to spread all the more extensively. Even when government measures were eventually implemented to encourage or require physical distancing, messages as to what was permitted were considered unclear, and early confusion reduced their effectiveness (Perrigo 2020). Beyond this, the abandonment of widespread testing was a further grave error. A test for the virus was developed by 10 January, but due to the UK adopting a centralized approach run out of Public Health England, without a view to make use of the hundreds of public and private laboratories across the country, the capacity for testing could not keep pace with the surge in cases (Talbot 2020). This made it impossible to follow international advice to use testing to trace and contain new cases of the virus.

These two key failures allowed the heightened transmission and lack of containment of the virus, contributing to the high number of cases. It has been suggested that, due to the paucity of testing at early stages, the case load in the first few months of the pandemic may have been almost twice what was reported—even compared to the second wave of cases that the UK (and other European countries) were facing in August, September and October—as many individuals (particularly those who are younger and in good health) are asymptomatic or only suffer mild symptoms and thus may be inclined not to self-report (Connors and Haughton 2020; Schraer 2020). Most significantly, however, it was the lack of resources and capacity which contributed to the high number of deaths. Moreover, these deaths were not spread evenly throughout the population, but rather focused on already vulnerable groups.

### Increased Vulnerability of Marginalized Groups

Failure to secure critical resources such as PPE and ventilators at an early stage left the UK at the back of the global queue for relevant trade deals. Global supplies of these resources dried up rapidly as countries competed, and when cases spiked, Britain’s failure to be well prepared became most evident. At times, this left patients without the care they needed, not to mention the staff working without protection in a high-risk environment. As such, a number of health professionals contracted the virus, though not all were symptomatic (Torjensen 2020). This meant they may have transmitted the virus to their patients, who were already in a vulnerable state.

As the UK did not move quickly to improve the capacity of its already underfunded healthcare system, it was necessary to introduce measures which would ease the burden on the NHS, rather than with a sole focus on the health of every individual. One of these was the discharge of elderly patients into care homes, without testing or quarantining, exposing some of the most vulnerable persons in our society to the virus. The Government was aware that age was a contributing factor to fatality rates, yet in an attempt to create capacity, it neglected those at greatest risk. It has become all too evident that many care homes do not have the resources necessary to keep their residents safe. Between 2 March and 12 June, almost 20,000 care home deaths were directly attributable to COVID-19, making up almost 50% of the total deaths in the UK (Office for National Statistics 2020a). Numerous analysts have also pointed to the impact of the pandemic on the BAME communities in the UK and in other countries, noting that the pandemic is disproportionately affecting these communities and those facing the worst of structural injustices, in particular those living in socially deprived areas of the country and without adequate access to healthcare (Mamluk and Jones 2020; Butcher and Massey 2020; Venkatapuram 2020). This has even been acknowledged by the UK Government itself in a report from Public Health England (Public Health England 2020a).

### Restrictions to Liberty

Lockdown in the UK lasted nearly 4 months, with varying degrees of restrictions between the nations. Arguably, because of the UK Government’s failure to exercise strong and competent leadership, particularly when the pandemic began to spread across Europe, lockdown had to extend over a longer period as case numbers continued to rise, rather than fall. This has caused millions to lose their jobs, seriously damaged the economy and, in itself, caused harm to individuals’ physical and mental health. Such a prolonged period of mandated cessation of free movement could have been avoided through a number of different strategies, as seen in other countries, such as strong contact tracing measures, as in East and Southeast Asia, or simply early lockdown and appropriate management of cases to ensure it did not become the widespread uncontrollable crisis that it did. Even now, however, as a result of weakness in the test and trace and the smartphone app, the UK—particularly in more densely populated England—is not succeeding in tracking all cases, which may lead to a second nationwide lockdown, or, as is now the case, sporadic, repeated need for localised ockdowns. Thus, while draconian restrictions of liberty have been shown not to be a necessary consequence of a pandemic when countries implement effective complementary strategies, the irony of the UK Government’s approach is that there will likely be the ongoing and pernicious threat of restrictions of liberty because of failures to manage the pandemic well.

All of this leads us to consider what impact this serial inaction and incompetent decision-making on the part of the UK Government in Westminster has had on core human rights and what lessons we might learn from the fiasco.

## Which Human Rights have been Implicated and What are the Governmental Duties to Prevent them?

The domain of human rights protection operates at international, European and domestic levels. Internationally, Article 12 of the International Covenant on Economic, Social and Cultural Rights (1966) recognizes the right of everyone to the enjoyment of the highest attainable standard of physical and mental health, and the UK is a signatory to this instrument albeit that it is not directly enforceable domestically. The relevance of this particular Article, and human rights more generally, has been well addressed elsewhere, especially as they relate to pandemics and preparedness assessed through the perspective of human rights (Eccleston-Turner and Brassington 2020; Gostin and Mason Meier 2020). Moreover, the Siracusa Principles on the Limitation and Derogation Provisions in the International Covenant on Civil and Political Rights, adopted by the UN Economic and Social Council in 1984, serve as an important benchmark against which to assess states’ action in times of emergency and to guard against violations of human rights in the name of addressing a crisis. We address their relevance to the current context below. For now, and primarily because of restriction of space, we confine the following discussion to the human rights that are directly enforceable in the UK, namely, those arising under the European Convention on Human Rights, as embodied in domestic law through the Human Rights Act 1998.

It is our contention that the UK Government’s inaction and failures, seen in the comparative light of international guidance and steps taken by governments both in other countries and even within the devolved administrations of the UK, have led to significant human rights implications and inequalities. As we have argued, in the UK’s case, the human rights concerns stem not from the specific legislative measures enacted in Westminster to combat the pandemic (which in our view have been proportionate and within the ambit of public health powers), but rather from the inaction and incompetence under which the measures were implemented and from the absence at times in implementing any measures at all.

Human rights are the basic rights and freedoms that belong to everyone, regardless of status. Human rights operate on the principle that, as Article 1 of the Universal Declaration of Human Rights declares, ‘All human beings are born free and equal in dignity and rights’. Human rights are clearly defined in several cornerstone normative instruments and are protected under British, European and international laws, including in the UK, under the Human Rights Act 1998, which incorporate into UK law the rights contained in the European Convention on Human Rights (ECHR). Human rights require that public authorities promote and protect the human rights of their citizens and the capability for citizens to flourish as citizens in a society. This obligation by the state includes, among other things, promoting and protecting rights to health and wellbeing. Accordingly, public authorities owe duties to citizens by ensuring that there is an adequate public health infrastructure that is marked by sufficient degrees of preparedness, prevention, containment and treatment, as tools to protect individuals who are most vulnerable.

Against the background of the response pandemic as outlined above, in principle—that is, what we would come to expect rather than what would come to fruition—the most affected human right is the right to life set out in Article 2 of the ECHR (‘Everyone’s right to life shall be protected by law’), and also reflected in Article 3 of the Universal Declaration of Human Rights (‘Everyone has the right to life, liberty and security of person’). This human right requires not only that the state not take the lives of its citizens except in those rare exceptions permitted under law but also that the state take positive action to protect lives. In the context of COVID-19, this would mean that the UK Government has an obligation to enact measures that seek to protect the population’s health from the virus. Indeed, the European Court of Human Rights (ECtHR) has found positive obligations to arise under Article 2 in a number of different contexts, including in the context of healthcare (*Calvelli and Ciglio v Italy*; *Vo v France*). Moreover, countries may also be under a positive obligation to prevent the spreading of contagious disease (*Poghosyan v Georgia, Ghavtadze v Georgia, Shelley v the United Kingdom*). For example, in *Shelley v the United Kingdom*, a case concerning a prisoner who complained that a decision to provide disinfecting tablets instead of needle exchange programmes failed to sufficiently address the risks caused by the sharing of infected needles, the ECtHR observed that ‘[…] it is not excluded that a positive obligation might arise to eradicate or prevent the spread of a particular disease or infection’.

To this end, we posit that Article 2 has been implicated by much of the UK’s *in*action, such as the failure to provide the proper protective equipment to healthcare staff, discharging potentially infected individuals into care homes, avoiding lockdown until weeks after other countries had done and even allowing the number of cases to reach such high levels by abandoning widespread test and trace. The evidence shows that the government allowed a high-risk virus to spread throughout the country without the proper means to protect citizens from the most severe effects. A CFR above 10% is unacceptable when contrasted to percentages of 1–2% in neighbouring countries with similar (or indeed) worse healthcare systems and infrastructures. Within the high mortality figures, the disparities in the affected persons, caused both directly and indirectly by governmental action, are in contravention of the government’s duties to promote equality. To this end, we note that the ECHR case of *Lopes de Sousa Fernandes v Portugal* has held that a country’s obligation to establish a regulatory framework for healthcare must be understood in a broad sense, which includes the duty to ensure the effective functioning of that regulatory framework, and thus encompass necessary measures to ensure implementation, including supervision and enforcement. Prof Kanstantsin Dzehtsiarou of the University of Liverpool has carefully analysed the implications of Article 2 ECHR. He argues that a court would find a violation of Article 2 if there was discriminatory treatment or flagrant denial of medical help (Dzehtsiarou 2020); in our view, there are grounds to make a claim that the UK’s failure to respond to the pandemic in March with proper PPE distributed across healthcare workers, robust lockdown early on, testing at wide scale for the virus and trace contacts effectively and testing elderly patients before discharging them from hospitals amounts to serious violations of UK citizens’ right to life.

Other ECHR human rights potentially, though less likely, implicated by the UK Government’s inaction and incompetence are the following:

- Article 3 (‘No one shall be subjected to torture or to inhuman or degrading treatment or punishment’)—which we note has no exculpatory provisions. Here, countries must refrain from treatment which damages a person’s physical health or causes them mental or psychological harm. Countries may also be obliged to take positive measures to protect the physical and mental health of individuals for whom it assumes special responsibility. In the context of COVID-19, denial of adequate medical treatment, such as access to a hospital, could trigger Article 3. As Dzehtsiarou (2020) observes, two criteria need to be satisfied here: the treatment or lack thereof needs to reach the minimal level of severity and the state’s involvement should be proved. This might be an uphill battle: ‘Proving that the state policy was a reason for overcrowded hospitals is a futile exercise at the ECtHR in times of pandemic’ (Dzehtsiarou 2020).
- Article 5 (‘Everyone has the right to liberty and security of person’). Here, as noted above, initial concern was raised that the legislative measures passed by the UK Government would unduly restrict one’s liberty. In other words, quarantine (or lockdown) could be an unjust deprivation of liberty. However, the exculpatory provision of Article 5(e)—‘the lawful detention of persons for the prevention of the spreading of infectious diseases’, and the conditions under which it should be done lawfully, viz. be made in accordance with national law, be limited in time and it should serve the purpose it is initiated for—namely, preventing spreading of infectious diseases, suggests that this human right would not be violated. The alternative argument—that lockdown has extended far beyond what was necessary and proportionate because of the government’s inability to get a handle on the virus—would also appear to be an uphill battle, largely because of the UK government’s laissez faire approach to movement restrictions. If anything, the government would be able to point to an *absence* of restrictions on liberty sufficient to trigger Article 5. Our point, however, is a concern with a protracted and prolonged period of lockdowns that will undoubtedly arise as a result of this uncoordinated and unprincipled approach. While this might not raise Article 5 concerns per se, it might return us to Article 3: does the de facto state policy of inaction ultimately amount to inhuman and degrading treatment for citizens left in a cycle of uncertainty and ever-present threat of restriction of movement?
- Article 8 (‘Everyone has the right to respect for his private and family life, his home and his correspondence’). This human right is certainly engaged by the government’s response to the pandemic; indeed, all people’s right to respect for private life around the world are implicated in times of pandemics, as governments seek to collect personal information concerning one’s health in an effort to contain and control the spread of a contagious disease. In the context of COVID-19, however, concerns have been raised about the smartphone apps and the sharing of confidential patient information between public authorities, particularly from doctors and public health officials to law enforcement agencies. Article 8 permits interference with one’s right to respect for private life, provided such interference is necessary and proportionate. Moreover, Article 8(2) permits interference where ‘[it] is in accordance with the law and is necessary in a democratic society […] for the protection of health […]’. It is beyond the scope of our article to detail the scope of this expansive human right, but the case law would suggest that a claim concerning Article 8 would have stronger grounds if based on allegations concerning widespread collection and sharing of confidential patient information or personal data (e.g. asking people to disclose their health and other personal information), rather than allegations that government policies to prevent the spread of COVID-19 interfered with one’s right to respect for private life. As far as we are aware, Article 8 has not yet been tested in a court of law in the context of a wider general preventive ground of an epidemic. This said, given the UK Government’s incompetent rollout of the smartphone COVID-19 tracing app and invocation of the notice provision under Regulation 3(4) of the Health Service Control of Patient Information Regulations 2002 to require organizations such as GP surgeries, NHS trusts and private healthcare organizations to disclose to other sectors and organizations (including private companies) confidential patient information for broadly defined ‘COVID-19 purposes’, concerns are certainly raised about undue interference with this human right (Secretary of State for Health and Social Care 2020).
- Article 11 (‘Everyone has the right to freedom of peaceful assembly and to freedom of association with others, including the right to form and to join trade unions for the protection of his interests’). This human right is experienced most pronouncedly in the ban many countries have enacted on large public gatherings to prevent the rapid spread of COVID-19 (e.g. no more or significantly reduced attendance in stadiums, concert halls, exhibition centres, theatres and so on). While in other countries, legitimate concerns may be raised that these measures might have been invoked to stifle political expression, in the UK, these measures are more likely seen as justifiable interference to prevent the spread of the virus. As with Article 8, however, any exceptions to Article 11 are only justifiable when they are necessary and proportionate. Sustained engagement with Article 11 rights because of ongoing governmental incompetence to deal effectively with the COVID-19 virus raises questions about how far the UK Government could continue to rely on the exceptions in an extended period of lockdowns and hand-to-mouth measures responding to new COVID-19 cases.

It is important to recognize that there have already been a number of Article 8 challenges to the lockdown—for example, *R (Dolan and Ors) v Secretary of State for Health and Social Care and Secretary of State for Education* [2020] EWHC 1786 (Admin)—but as we have argued, the initial measures in the first 6 months of the pandemic arguably may be seen as necessary and proportionate in light of the severity of the virus; in other words, these fall within the exculpatory provisions seen in the famous ‘paragraph 2’ of human rights (Laurie 2020). However, our contention is that faster, more decisive and more robust action to prevent rapid spread and in turn the high number of cases could have enabled a nearly 4-month lockdown to be shorter; moreover, these failures will likely extend the period of uncertainty and future lockdowns, whether these are on a local, regional or national basis. Most importantly, capacity for mass testing and means of tracking and tracing instances of the virus would have enabled better containment, as demonstrated in South Asia and Australasia, and as such shorter lockdown periods. Even now as the UK attempts to avoid national lockdown, there are insufficient measures in place to ensure that further outbreaks are unlikely. It will not be surprising to see a repeated rise in ‘hotspot’ lockdowns over the coming months and longer, for as long as international travel is permitted and neither an effective vaccine nor treatment are available.

From the legal standpoint, it must be recognised that the UK has certainly acted to enact enabling legislation with respect to its response to COVID-19. Notable examples are the Health Protection (Coronavirus, Restrictions) (England) Regulations 2020, and equivalent measures coming from the devolved administrations, and—in September 2020—the Health Protection (Coronavirus, Restrictions) (No. 2) (England) (Amendment) (No. 4) Regulations 2020. However, these last provisions were published only minutes before the Act came into force, allowing no time for appropriate scrutiny. There are multiple human rights objections that can be raised to such legislative moves. For example, concerning the first set of regulations, there was no requirement for the Secretary of State to have regard to the rights and freedoms that the provisions restricted. As to the second example, due process alone requires adequate opportunity for scrutiny of any law that has as its objective the curtailment of citizens’ rights and freedoms. Indeed, colleagues have written elsewhere about the myriad ways in which such provisions—as positive empowerment of the state to act—are violations of human rights (Hoar 2020). In contrast, and to continue with our central theme of violation through inaction, we contend that even these attempts to deploy law in the face of the pandemic are, in fact, egregious examples of failures in pandemic preparedness and due respect for human rights. This is because the legislation itself is a further evidence of inaction on the part of the UK Government relative to what is required under human right laws.

In making this argument, we rely on the international Siracusa Principles, mentioned above. These make specific provision in the context of public health, notably that measures responding to an emergency must be:

1. Provided for and carried out in accordance with law
2. Directed toward a legitimate objective of general interest
3. Strictly necessary in a democratic society to achieve the objective
4. The least intrusive and restrictive to achieve the objective
5. Based on scientific evidence and neither arbitrary nor discriminatory in application
6. Of limited duration, respectful of human dignity and subject to review

This reflects the general requirement for proportionality of response in all matters, which in turn requires transparent explanation and justification of all measures undertaken through law. Yet, the September 2020 Regulations merely contain a bare statement in the Introductory Text to the effect ‘The Secretary of State considers that the restrictions and requirements imposed by [The Regulations] are proportionate to what they seek to achieve, which is a public health response to that threat’. The lack of parliamentary time to assess this claim falls foul of both the letter and the spirit of the Siracusa Principles. Furthermore, in the Explanatory Note, it is stated: ‘No impact assessment has been carried out for these Regulations’. This is in direct contradiction to Principle 4. The notion of ‘in accordance with law’ stated in Principle 1 is well recognized in the human rights field to include the ‘knowability’ of the law. Once again, the lack of transparency in the production of these laws suggests a serious breach. And finally, as to Principle 6, the requirement that laws be ‘subject to review’ can be assessed by the UK Government’s own benchmark criteria made public on 16 April 2020. These were drafted after a review of the lockdown to that date:

- That the NHS is able to cope
- A ‘sustained and consistent’ fall in the daily death rate
- Reliable data showing the rate of infection was decreasing to ‘manageable levels’
- That the supply of tests and PPE could meet future demand
- That the government could be confident that any adjustments would not risk a second peak (BBC 2020d)

While these criteria were used to defend an extension of lockdown, the repeated legislative initiatives since then suggest that unless and until criteria such as these are met, regulations of the kind discussed here will continue to be promulgated. Yet, it is precisely because of an uncertain and disputed scientific basis, the chaos of track and trace in the UK and the sustained and unacceptable death rate that these criteria will remain elusive. Thus, violation is heaped upon violation. By any human rights standard, the UK has failed its citizens on an unconscionable scale.

## Conclusion

The UK’s response to the pandemic has lagged far behind that of other countries and international guidance. Despite being forewarned in early January by the WHO of the potentially severe consequences of the virus, the UK was not forearmed, which later led to shortages in resources when they were most critically needed. The UK’s delayed response, implementing physical distancing measures much later than its neighbours in Europe (and across much of the globe), allowed case numbers to increase exponentially, placing greater numbers of its citizens’ lives at risk. Its weaknesses and failures in planning to test, track and trace both at the start and as it attempts to exit lockdown continue to place its citizens’ lives at risk, particularly those who are already vulnerable. The implications of these failures are all too clear, when compared to the number of cases, deaths and fatality rates worldwide. Rather than delaying the inevitable, the UK Government ought to have responded better to the pandemic and, in doing so, avoided the serious human right implications and likely unjustifiable infringements that have occurred.

Through all this, we offer three lessons that should be learnt from the UK Government’s response to COVID-19:

1. *Failing to prepare means you prepare to fail*. Foremost, what we have seen is a failure by the UK Government to prepare for COVID-19, both in terms of anticipatory measures and during the response phase of the pandemic. While this specific pandemic could have not been foreseen, a pandemic certainly was a ‘known unknown’—something that had a reasonable chance of occurring in the near future. Moreover, the UK Government had ample time to prepare in the 2 months preceding the wave that hit the country in March 2020—the evidence was incontrovertible from the experiences of other countries. The UK acted too late and too haphazardly. Relatedly, the UK Government failed to heed the clear findings and recommendations from the Exercise Cygnus simulation run in 2016 to ensure sufficient amount of PPE was available and distributed across the country and more generally to ensure the capability and capacity to surge resources into key areas.
2. *Over-centralisation can perpetuate risk of failure*. In the UK’s case, relying solely on Public Health England—which had 290 people across the entire country—to coordinate testing and tracing early on undoubtedly has led to thousands of cases going untested, not to mention very likely a considerable number of unnecessary deaths. As *The Economist* (2020a) has noted: ‘Complaints about centralisation persist. Local authorities are struggling to get data from NHS Test and Trace. According to Leicester’s mayor, Sir Peter Soulsby, the city’s recent outbreak was exacerbated by poor-quality data and delays before they were provided. They are, he says, still too slow to arrive—the last batch came [two weeks prior]—and they identify cases only at a postcode level, without addresses or workplaces, and with ethnicity for only a minority of cases’. It is telling that the putative “United” Kingdom has fragmented into its constituent countries as the devolved administrations realized that the UK Westminster response was not serving the interests of their own citizens. Additionally, it is far from clear that the UK Government’s response to Public Health England’s alleged failings in coordination—the creation in August 2020 of a new National Institute for Health Protection (and an uncertain future for Public Health England)—will necessarily resolve the challenges associated with the UK’s centralized system of governance and government. Indeed, given that the NIHP aims to bring together Public Health England, NHS Test and Trace and the Joint Biosecurity Centre, this actually suggests that the Government remains committed to centralize decision-making and operate top-down.
3. *Contrary to anticipated concerns about draconian government action, future preparedness for pandemics must take account of likely governmental inaction and incompetence*. In our view, there are serious concerns about potential infringement of core human rights, most particularly Article 2 ECHR. In assessing the UK Government’s response to COVID-19, people’s right to life—especially those who are most vulnerable—has manifestly been affected in the worst ways possible. The UK Government failed to uphold its positive obligations owing to its citizens to ensure PPE was available for frontline healthcare workers, testing capacity and contact tracing could be surged when the virus became an epidemic in the country and that no patients would be discharged from hospitals back into the community without first testing them for the virus.

We sincerely hope that this analysis brings further strength and support to the calls that the UK Government launch an independent inquiry into what went wrong, to prevent such an outcome from ever happening again. Sadly, as the pandemic looks set to continue for an uncertain amount of time, there remain myriad lessons to be learned about what it means to show due respect for human rights especially as matters may well become worse before they become better.

## Footnotes

1 Unless otherwise stated, all data presented is reflective of the situation as of 1 October 2020

2 A detailed timeline of the UK’s response, alongside international guidance and other countries actions, may be found at https://www.bbc.co.uk/news/world-europe-51876784
